# A multicenter phase II study of bendamustine, rituximab, and cytarabine (BRAC) for relapsed or refractory patients with follicular lymphoma or mantle cell lymphoma

**DOI:** 10.1186/s40164-022-00264-3

**Published:** 2022-02-25

**Authors:** Nobuhiko Nakamura, Senji Kasahara, Junichi Kitagawa, Hiroshi Nakamura, Michio Sawada, Kenji Fukuno, Yuhei Shibata, Yuto Kaneda, Takeshi Hara, Nobuhiro Kanemura, Hisashi Tsurumi, Masahito Shimizu

**Affiliations:** 1 Department of Hematology and Infectious Disease, Gifu University Hospital, 1-1, Yanagido, Gifu, 501-1194 Japan; 2 Department of Hematology, Gifu Municipal Hospital, Gifu, Japan; 3 Department of Hematology, Gifu Red Cross Hospital, Gifu, Japan; 4 Department of Hematology, Takayama Red Cross Hospital, Gifu, Japan; 5 Department of Hematology, Matsunami General Hospital, Gifu, Japan

**Keywords:** Follicular lymphoma, Mantle cell lymphoma, Bendamustine, Rituximab, Cytarabine

## Abstract

This phase II clinical trial aimed to evaluate the efficacy and safety of the combination therapy of bendamustine, cytarabine, and rituximab (BRAC) in patients with relapsed or refractory follicular lymphoma (FL) or mantle cell lymphoma (MCL). Thirteen patients were enrolled and received a median of 4 cycles (range 2–6) of BRAC. The complete response rate was 61.5%, and the overall response rate was 84.6%; the 2-year overall survival was 76.9%, and the 2-year progression-free survival was 69.2%. Although all patients received G-CSF prophylaxis, grade 3 or higher neutropenia was observed in all cycles, and the incidence of febrile neutropenia was 20%. Grade 4 thrombocytopenia was observed in 92.5% of all cycles, and platelet transfusion was performed in 94%. Although hematological toxicity was relatively high, BRAC therapy was effective for relapsed and refractory FL or MCL. Further studies are needed to determine the optimal dose of BRAC therapy.

*Trial registration* The UMIN Clinical Trials Registry, UMIN000009797. Registered 17 January 2013, https://upload.umin.ac.jp/cgi-open-bin/ctr/ctr_view.cgi?recptno=R000011103

**Supplementary Information:**

The online version contains supplementary material available at 10.1186/s40164-022-00264-3.

## To the Editor

The combination of rituximab and chemotherapy has significantly improved outcomes for B-cell non-Hodgkin lymphoma (NHL) patients. However, conventional therapies cannot cure follicular lymphoma (FL) and mantle cell lymphoma (MCL) [1, 2]. Bendamustine has demonstrated high efficacy as monotherapy and in combination with rituximab for relapsed or refractory indolent B-cell NHL and MCL [3]. Cytarabine has been reported to be synergistic with bendamustine and is a new candidate for combination therapy [4]. Results of three-drug combination therapy of bendamustine, cytarabine, and rituximab (BRAC) in patients with MCL patients conducted overseas have shown high efficacy and safety [5, 6]. Although no validated phase III comparative study has yet been conducted, bendamustine and cytarabine combination therapy may be a promising treatment regimen. Therefore, we conducted a phase II clinical trial to evaluate efficacy and safety of BRAC. Due to slow accrual, the study was prematurely closed with a total of only 13 patients.

A multicenter, open-label, single-arm, phase II clinical trial was conducted in 3 hospitals from the Gifu Hematology Study Group in Japan. Patients were eligible if they were aged 20–80 years, Eastern Cooperative Oncology Group performance status of 0–2 and had histologically confirmed, relapsed or refractory FL or MCL according to the WHO classification [7]. Patients with histological transformation to a higher degree of lymphoma have been ruled out before the initiation of BRAC treatment. All patients received BRAC (rituximab 375 mg/m^2^ on day 1; bendamustine 90 mg/m^2^ over 30–60 min on days 2 and 3; cytarabine 600 mg/m^2^ over a 2-h infusion on days 2–4; all administered intravenously) every 4 weeks for up to 6 cycles. Prophylaxis with G-CSF (filgrastim 50 μg/m^2^) was given 24 h after the last cytarabine dose in every cycle. If grade 3 or higher non-hematologic toxicity was observed, the dose of bendamustine was reduced from 90 mg/m^2^ to 60 mg/m^2^ in the subsequent cycles. The dose of rituximab and cytarabine was not reduced. The primary endpoint was the complete response (CR) rate. The response was evaluated according to the Cheson criteria, 2007 [8]. The CR rate for BR therapy in patients with relapsed or refractory indolent B-cell NHL and MCL is 54–59% [9]. In addition, the CR rate for BRAC therapy in relapsed MCL is 70% [5]. Therefore, we set the expected CR rate at 70% and the threshold CR rate at 45%, one-sided α error of 0.05, and β error of 0.2, and the minimum number of patients required at 24. We assumed that 10% of the enrolled patients would be ineligible, and the sample size was set at 27.

Though enrollment of 27 patients was planned, 13 patients were enrolled from May 2013 to May 2018. The demographics and characteristics of patients are shown in Table 1. The median age was 65 (53–73) years, and 6 patients (46.2%) were male. The histological types were FL in 9 patients and MCL in 4 patients. The median number of previous treatment regimens was 1 (range: 1–6), and 3 patients (23.1%) were refractory to their last chemotherapy. All patients had received rituximab-containing chemotherapy, four patients had been treated with bendamustine, two patients had been treated with cytarabine, and none had been treated with Bruton’s tyrosine kinase (BTK) inhibitor. Patients received a median of 4 cycles (range 2–6) of BRAC. Seven of 13 patients (53.8%) were able to complete the scheduled treatment. One patient received an autologous hematopoietic stem-cell transplantation (HSCT) after 4 cycles, one patient received an allogeneic HSCT after 3 cycles, and no patient received BTK inhibitor maintenance. The CR rate was 61.5% [95% confidence interval (CI) 31.6–86.1], and the overall response (OR) rate was 84.6% (95% CI 54.6–86.1). In 9 patients with FL, the CR rate was 66.7%, and the OR rate was 100%. The median follow-up for 10 survivors from enrolment was 31.2 months (range 20.8–39.6 months). The 2-year overall survival (OS) was 76.9% (95% CI 44.2–91.9%) [Additional file 1: Figure S1(A)], and the 2-year progression-free survival (PFS) was 69.2% (95% CI 37.3–87.2%) [Additional file 1: Figure S1(B)].

**Table 1:** Patients demographics and characteristics

Patient	Age, gender	Diagnosis	Previous regimen	Cycles of BRAC	Response	Treatment after BRAC
1	61, F	FL G1	R-CHOP	5	PR	–
2	53, F	FL G3b	R-CHOP	4	CR	auto HSCT
3	71, F	FL G2	R-THP-COP, R-IMVP-16/CBDCA, R-B, CHASER	4	CR	–
4	66, M	FL G1	R-THP-COP	6	PR	–
5	63, F	FL G2	R-CHOP	4	CR	–
6	61, M	MCL	R-HyperCVAD/MA	3	CR	–
7	67, M	MCL	R-CHOP, R-IMVP-16/CBDCA, CHASER, R-HyperCVAD/MA	2	NA	–
8	57, M	FL G2	R-CHOP, R-B	3	PR	allo HSCT
9	69, F	FL G2	R-THP-COP, R-B, R, R-Flu, GCD-R, Ibritumomab Tiuxetan	3	CR	–
10	72, M	MCL	R-CHOP, R-B	2	NA	–
11	72, M	FL G2	R-CHOP, R-B	4	CR	–
12	65, F	FL G1	R-CHOP	4	CR	–
13	69, F	MCL	R-CHOP	6	CR	–

FL: follicular lymphoma; G: grade; MCL: mantle cell lymphoma; R-CHOP: rituximab, cyclophosphamide, doxorubicin, vincristine, and prednisolone; R-THP-COP: rituximab, cyclophosphamide, pirarubicine, vincristine, and prednisolone; R-IMVP-16/CBDCA: rituximab, ifosfamide, methotrexate, etoposide, carboplatin, and methylprednisolone; R-B: rituximab, bendamustine; CHASER: cyclophosphamide, high dose cytarabine, dexamethasone, etoposide, and rituximab; R-HyperCVAD/MA: rituximab, hyperfractionated cyclophosphamide, vincristine, doxorubicin, and dexamethasone, alternating with high dose methotrexate and cytarabine; R: rituximab; R-Flu: rituximab, fludarabine; GCD-R: gemcitabine, carboplatin, dexamethasone, and rituximab; PR: partial response; CR: complete response; NA: not applicable; HSCT: hematopoietic stem cell transplantation

Fifty cycles of BRAC were given. Table 2 summarizes the hematological and non-hematological toxicities. Although all patients received G-CSF prophylaxis, grade 3 or higher neutropenia was observed in all cycles, and the incidence of febrile neutropenia was 20%. Grade 4 thrombocytopenia was observed in 92.5% of all cycles, and platelet transfusion was performed in 47 of 50 cycles (94%). Treatment initiation was delayed in 7 of 13 patients (53.8%), primarily due to treatment toxicity. The dose of bendamustine was reduced in 6 of 50 patient cycles (12.0%).

**Table 2:** Treatment cycles with hematological toxicities and non-hematological toxicities (50 patient-cycles)

Toxicity, n (%)	Grade 3	Grade 4
Hematologic		
Neutropenia	5 (10.0)	41 (82.0)
Anemia	39 (78.0)	1 (2.0)
Thrombocytopenia	4 (8.0)	46 (92.0)
Non-hematologic		
Sepsis*	0 (0.0)	1 (2.0)
Febrile neutropenia	10 (20.0)	0 (0.0)
Cytomegalovirus infection	2 (4.0)	0 (0.0)
Hypokalemia	1 (2.0)	0 (0.0)
Hemothorax	0 (0.0)	1 (2.0)
Hyperamylasemia	2 (4.0)	0 (0.0)

^*^The causative organism of sepsis was Bacillus spp

In the present study, the efficacy and safety of BRAC therapy for relapsed or refractory FL or MCL were evaluated. A good response rate (CR rate: 61.5%, OR rate: 84.6%) was seen. In particular, this is the first study to confirm the response to BRAC therapy of relapsed or refractory FL (CR rate: 66.7%, OR rate: 100%). On the other hand, hematological toxicity was relatively high, with 20% (10/50 cycles) having febrile neutropenia and the need for platelet transfusion in almost all cycles. Visco et al. reported the use of R-BAC (rituximab 375 mg/m^2^, bendamustine 70 mg/m^2^, cytarabine 800 mg/m^2^) in elderly patients with previously untreated or relapsed or refractory MCL [5], and they reduced the dose of cytarabine to 500 mg/m^2^ in the next study because of high hematological toxicity [6]. Unfortunately, sufficient conclusions could not be drawn because the present study closed prematurely due to slow accrual, but it was possible to show that BRAC therapy might be useful, especially for FL. The reason for the slow accrual is uncertain, but one reason may have been the emergence of new drugs. For example, obinutuzumab and lenalidomide were approved for the treatment of FL, and bortezomib and ibrutinib were approved for the treatment of MCL in Japan during the study period which may have increased the number of treatment options and slowed patient enrollment. BTK inhibitors, such as ibrutinib, have been reported to be synergistic with rituximab [10] and are effective agents against MCL. Furthermore, the usefulness of anti-CD19 chimeric antigen receptor (CAR) T-cell therapy for FL and MCL has been recently reported [11, 12]. Although administration of BRAC before leukocyte apheresis should be avoided because bendamustine reduces the efficiency of lymphocyte collection, BRAC may be effective as bridging chemotherapy to CAR-T. Further studies are needed to determine the optimal dose and timing of BRAC therapy.

## Supplementary Information

**Additional file 1: Figure. S1 **Overall survival (**A**) and progression-free survival (**B**) for all patients (n = 13). Overall survival (**C**) and progression-free survival (**D**) for follicular lymphoma (FL) (n = 9) and mantle cell lymphoma (n = 4).

## Data Availability

The datasets used and/or analysed during the current study are available from the corresponding author on reasonable request.

## Abbreviations

NHL: Non-Hodgkin lymphoma
FL: Follicular lymphoma
MCL: Mantle cell lymphoma
CR: Complete response
PR: Partial response
OR: Overall response
OS: Overall survival
PFS: Progression-free survival
CI: Confidence interval
BTK: Bruton’s tyrosine kinase
CAR: Chimeric antigen receptor

## References

[1] Hanel W, Epperla N (2021). Evolving therapeutic landscape in follicular lymphoma: a look at emerging and investigational therapies. J Hematol Oncol.

[2] Ladha A, Zhao J, Epner EM, Pu JJ (2019). Mantle cell lymphoma and its management: where are we now?. Exp Hematol Oncol.

[3] Rummel M, Kaiser U, Balser C (2016). Bendamustine plus rituximab versus fludarabine plus rituximab for patients with relapsed indolent and mantle-cell lymphomas: a multicentre, randomised, open-label, non-inferiority phase 3 trial. Lancet Oncol.

[4] Castegnaro S, Visco C, Chieregato K (2012). Cytosine arabinoside potentiates the apoptotic effect of bendamustine on several B- and T-cell leukemia/lymphoma cells and cell lines. Leuk Lymphoma.

[5] Visco C, Finotto S, Zambello R (2013). Combination of rituximab, bendamustine, and cytarabine for patients with mantle-cell non-Hodgkin lymphoma ineligible for intensive regimens or autologous transplantation. J Clin Oncol.

[6] Visco C, Chiappella A, Nassi L (2017). Rituximab, bendamustine, and low-dose cytarabine as induction therapy in elderly patients with mantle cell lymphoma: a multicentre, phase 2 trial from Fondazione Italiana Linfomi. Lancet Haematol.

[7] Swerdlow SH, Campo E, Pileri SA (2016). The 2016 revision of the World Health Organization classification of lymphoid neoplasms. Blood.

[8] Cheson BD, Pfistner B, Juweid ME (2007). Revised response criteria for malignant lymphoma. J Clin Oncol.

[9] Robinson KS, Williams ME, van der Jagt RH (2008). Phase II multicenter study of bendamustine plus rituximab in patients with relapsed indolent B-cell and mantle cell non-Hodgkin’s lymphoma. J Clin Oncol.

[10] Albertsson-Lindblad A, Freiburghaus C, Jerkeman M, Ek S (2019). Ibrutinib inhibits antibody dependent cellular cytotoxicity induced by rituximab or obinutuzumab in MCL cell lines, not overcome by addition of lenalidomide. Exp Hematol Oncol.

[11] Hirayama AV, Gauthier J, Hay KA (2019). High rate of durable complete remission in follicular lymphoma after CD19 CAR-T cell immunotherapy. Blood.

[12] Wang M, Munoz J, Goy A (2020). KTE-X19 CAR T-cell therapy in relapsed or refractory mantle-cell lymphoma. N Engl J Med.

