# Supplementary figures and images for: A multicenter phase II study of bendamustine, rituximab, and cytarabine (BRAC) for relapsed or refractory patients with follicular lymphoma or mantle cell lymphoma
Source: Exp Hematol Oncol. 2022 Feb 25;11:9. doi: 10.1186/s40164-022-00264-3 (PMC8876747; doi:10.1186/s40164-022-00264-3)

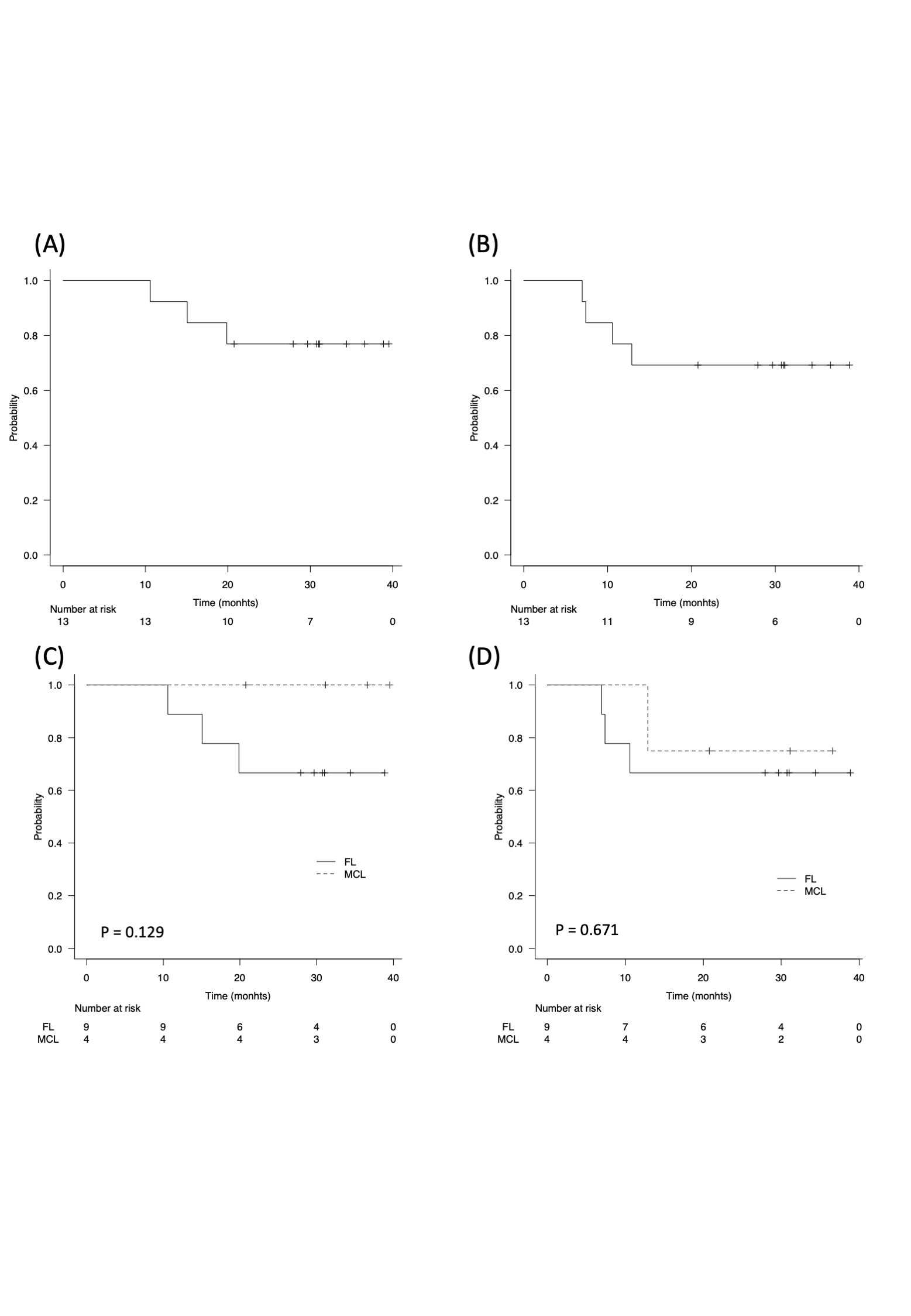

Supplement: Supplementary file 1 — Additional file 1: Figure. S1 Overall survival (A) and progression-free survival (B) for all patients (n = 13). Overall survival (C) and progression-free survival (D) for follicular lymphoma (FL) (n = 9) and mantle cell lymphoma (n = 4). [file 40164_2022_264_MOESM1_ESM.tiff]
